# Establishment of a doxycycline-regulated cell line with inducible, doubly-stable expression of the wild-type p53 gene from p53-deleted hepatocellular carcinoma cells

**DOI:** 10.1186/1475-2867-5-27

**Published:** 2005-08-23

**Authors:** Tian-Yi Chi, George G Chen, Lok-Kee Ho, Paul BS Lai

**Affiliations:** 1 Department of Surgery, Chinese University of Hong Kong, Prince of Wales Hospital, Shatin, New Territories, Hong Kong SAR, China

**Keywords:** hepatocellular carcinoma, p53, tetracycline, doxycycline, gene expression.

## Abstract

p53 is important in the development of hepatocellular carcinoma (HCC) and in therapeutic approaches, but the mechanism whereby it inhibits HCC growth is still unclear. The aim of the present study was to establish a HCC cell system in which p53 levels can be regulated. Full-length wild-type p53 cDNA obtained by PCR was cloned into a retroviral response vector controlled by the tetracycline responsive element (RevTRE-p53). The regulatory vectors RevTet-Off and RevTRE-p53 were transfected into a packaging cell line, PT67. Hep3B cells in which the p53 gene was deleted were infected with RevTet-Off viral particles from the PT67. Three G418-resistant cell clones with high luciferase expression and low background were infected with RevTRE-p53. By screening dozens of RevTRE-p53-infected clones with hygromycin we identified the one with the highest expression of p53 and the lowest background after doxycycline treatment. The results showed that p53 expression in this cell clone could be simply turned on or off by removing or adding doxycycline. Furthermore, it was found that the level of p53 protein was negatively and sensitively related to the doxycycline concentration. In conclusion, we have established a HCC cell line in which p53 expression can be switched on or off and regulated in a dose- and time-dependent manner.

## Introduction

Hepatocellular carcinoma (HCC) is one of the most common malignancies worldwide and is estimated to cause over one million deaths every year [1]. Risk factors have been identified, including chronic infection with hepatitis B and C viruses and exposure to aflatoxin B1, but the exact molecular mechanisms of carcinogenesis have not been completely defined. Mutation or deletion of the p53 gene, which plays an important role in cell growth, division and apoptosis by acting as a transcription factor or by forming complexes with other proteins [2], are frequently detected in HCC [3]. In addition, loss of the functional p53 gene is associated with lower cellular differentiation and poor prognosis [4]. Hence, restoration of a wild-type p53 gene is an attractive approach to the treatment of HCC [5].

Tight quantitative and temporal control of gene expression is very useful for basic biological and medical research applications. Several inducible gene expression systems have been developed such as hormones, heavy metal ions and heat shock [6]. However, most of them are limited by low and nonspecific induction, toxic effects of the inducing agents, high basal levels of gene expression under non-induced conditions and pleiotropic effects [7]. The tetracycline (tet)-regulated gene expression system overcomes many of the problems of other inducible systems [8,9] and has been widely utilized in mammalian cell culture [10], transgenic mice [11] and other species [12]. Furthermore, the retrovirus-mediated tet-regulated gene expression system (RevTet-Off/On) combines the advantages of retroviral transfer with those of tet-regulation and allows faster and more efficient establishment of regulated gene expression in a wide variety of cell types [13]. In the RevTet-Off system, transcription of the gene of interest is turned off in the presence of tetracycline or doxycycline (a derivative of tetracycline). In contrast, transcription of this gene is turned on in the presence of tetracycline or doxycycline in the RevTet-On system.

Loss of p53 function by mutation not only results in the malignant transformation of cells but also enhances resistance to anticancer drugs and radiation [14]. Studies on human tumors carrying mutated or deleted p53 show that the introduction of exogenous wild-type (wt) p53 could lead to apoptotic death of the tumor cells and effectively inhibit their growth [15]. It is therefore important to gain better insight into p53 function in HCC. We therefore cloned the wt p53 gene from a human hepatoblastoma cell line, HepG2, into a human hepatocellular cell line, Hep3B, using a retrovirus-mediated tet-regulated gene expression system (RevTet-Off) and established a HCC cell line with tightly controllable, doubly-stable expression of the wt p53 gene.

## Materials and methods

### RevTet-Off System

The RevTet-Off System was purchased from Clontech Laboratories (Palo Alto, CA). It is a complete retroviral gene expression system including a retroviral regulatory tTA vector (pRevTet-Off), a retroviral response vector (pRevTRE), a control vector (pRevTRE-Luc) and a packaging cell line (PT67).

### Cell lines and culture

Hep3B and HepG2 cells were purchased from the American Type Culture Collection. The Hep3B cells were derived from a human HCC deficient in p53 [16], the HepG2 cells from a human hepatoblastoma expressing wt p53 [17]. PT67 packaging cells were derived from an NH/3H3-based line expressing the 10A1 virus envelope [18]. All cells were grown in Dulbecco's modified Eagles's minimal essential medium with high glucose (Invitrogen, Carlsbad, California) supplemented with 10% fetal bovine serum (Clontech, Palo Alto, CA) and incubated at 37°C in a humidified atmosphere containing 95% air and 5% carbon dioxide.

### Reagents

G418 (Clontech, Palo Alto, CA) was prepared as a 10 mg/ml stock solution. Hygromycin B was available from Clontech as a stock solution (50 mg/ml). Both were stored at 4°C. Aliquots of 1 mg/ml Doxycyclin (Clontech, Palo Alto, CA) were stored in the dark at -20°C.

### Construction of the RevTRE-p53 plasmid

The full-length cDNA of wt p53 was obtained from the HepG2 cells by RT-PCR. The PCR primers were as follows: 5'-TTA AGC TTT TTG CGT TCG GGC TGG GAG C-3' and 5'-CGA TCG ATA TGG TGG CAT GAA CCT GTG G-3', which contained restriction sites for *Hind *III and *Cal *I respectively. The resulting PCR products were further purified with a QIAquick gel extraction kit (Qiagen, Valencia, CA) according to the manufacturer's instructions. The full-length p53 cDNA was cloned into the pRevTER vector using the restriction enzymes *Hind *III/*Cla *I.

### Transformation, purification and identification of retrovirus plasmids

The retrovirus vectors provided in the RevTet-Off System and recombinant pRevTRE-p53 plasmid were transformed into *E. coli *(DH5α). Plasmid DNA was purified using a QIAprep mini kit (Qiagen, Valencia, CA) according to the manufacturer's instructions. The purified plasmids were digested with the restriction enzymes *Hind III *and *Cla *I (Invitrogen, Carlsbad, California) to confirm the insert and the vector.

### Sequence analysis of the recombinant pRevTRE-p53 plasmid

The purified pRevTRE-p53 plasmid was sequenced with an ABI PRISM BigDye Terminator Cycle Sequencing kit (PE Applied Biosystems, Foster City, CA) according to the manufacturer's protocol. A pair of primers were designed for sequencing pRevTRE-p53: 5'-AAG CTT TTT GCG TTC GGG CTG GGA GC-3' and 5'-ATC GAT ATG GTG GCA TGA ACC TGT GG-3'. Recombinant pRevTRE-p53 plasmid was purified from *E. coli *with a QIAGEN Plasmid Midi kit (Qiagen, Valencia, CA).

### Package of retroviral plasmids and recombinant pRevTRE-p53 plasmid

PT67 cells were transfected with the pRevTet-Off, pRevTRE-Luc or pRevTRE-p53 plasmids using Lipofectamine Reagent (Invitrogen, Carlsbad, California) according to the manufacturer's instructions. The transfected cells were selected in 600 μg/ml G418 or 400 μg/ml hygromycin for 2 weeks and the selective media were replaced every 4 days. Well-separated antibiotic-resistant clones were individually picked with cloning discs and transferred to 24-well plates in selective medium. The cells were then transferred to larger culture vessels before confluence, and aliquots of early passages of cells (1 × 10^6^) were frozen in liquid nitrogen.

### Determination of viral titer

The supernatant from virus-containing PT67 cells was filtered through a 0.45 μm filter and added to Hep3B cells in the presence of 4 μg/ml polybrene (Sigma Chemical Co. St. Louis, MO). To determine the viral titer, infection of six 10-fold serial dilutions was performed in 6-well plates. The medium was replaced 6 h after infection. Three serial infections were performed. Forty-eight hours after infection, Hep3B cells were subjected to selection by G418 (0.5 mg/ml) or hygromycin (0.05 mg/ml) for one week. The viral titer corresponded to the number of colonies developed at the highest dilution.

### Infection of Hep3B cells by RevTet-Off virus

Hep3B cells were infected with supernatant from fresh PT67 cells containing RevTet-Off virus and selected with G418 as described above. The isolated G418-resistant clones were transferred to a 24-well plate with cloning cylinders. Before confluence, the cells were transferred into larger culture vessels.

### Screening for inducible G418-resistant clones by RevTRE-Luc virus

The pRevTRE-Luc control vector was constructed by cloning the luciferase gene into the *Hind *III/*Cal *I sites in pRevTRE. G418-resistant clones infected with RevTRE-Luc virus were selected by screening luciferase expression. For each clone, two additional cultures in wells of a 6-well plate were deployed as a test plate for screening. Infection by the RevTRE-Luc virus was performed as described previously. Twenty-two hours after infection, 1 μg/ml doxycycline (Dox) was added to one of the two test wells. Luciferase activity was analyzed using a luciferase assay system kit (Promega Corporation, Madison) according to manufacturer's instructions.

### Infection of G418-resistant clones by RevTRE-p53 virus

The selected G418-resistant clones were infected with supernatant from PT67 cells containing RevTRE-p53 virus. The clones were selected with hygromycin for two weeks as described previously. Healthy hygromycin-resistant clones were individually transferred to a 24-well plate with cloning cylinders and propagation was continued in selective medium. Aliquots of early passages of hygromycin-resistant cells (1 × 10^6^) were frozen in liquid nitrogen.

### Reverse transcription-polymerase chain reaction (RT-PCR) analysis

Total RNA was extracted from cells using a RNeasy Mini kit (Qiagen, Valencia, CA) according to the manufacturer's instructions and reverse-transcribed to cDNA with a First-Strand cDNA Systhesis Kit (Amersham Biosciences, Selangor Darul Ehsan, Malaysia). The neomycin gene and p53 cDNA were amplified by PCR, generating a 382-bp fragment of the neomycin gene (forward primer: 5'-TCC TGT CAT CTC ACC TTG CTC C-3' and reverse primer: 5'-GCA ATA TCA CGG GTA GCC AAC G-3'); a 282-bp cDNA fragment of p53 (5'-TAC ATG TGT TAA CAG TTC CTG CA-3' and 5'-TTC TGA CAA CGA TCG GAG GA-3'), or a 114-bp fragment of β-microglobulin gene (5'-ACC CCC ACT GAA AAA GAT GA-3' and 5'-ATC TTC AAA CCT CCA TGA TG-3') that served as an internal standard for RNA integrity and equal gel loading. Thermocycling was carried out at 94°C for 3 min and 30 amplification cycles of 94°C for 30 s, 62°C for 30 s and 72°C for 1 min, followed by 72°C for 7 min. The PCR products were electrophoresed in a 1.2% agarose gel and the cDNA fragments were stained with ethidium bromide and visualized under an Ultraviolet Transilluminator (UVP Inc.Upland, CA).

### Western blot

Cells were lysed in extraction buffer and 20 μg/ml of protein were subjected to 12% SDS-polyacrylamide gel electrophoresis. The proteins were transferred electrophoretically on to nitrocellulose membranes (Amersham, Piscataway, NJ). The membranes were probed with a monoclonal mouse anti-human p53 antibody (1:1000) (Santa Cruz, CA) and β-actin (1:1500) (Santa Cruz, CA) and were then incubated with goat anti-mouse IgG-HRP antibody (1:2000) (Santa Cruz, CA). The signals were detected using an enhanced chemiluminescence system.

## Results

### Identification of recombinant RevTRE-p53 plasmids by restriction enzymes

**Figure 1:**
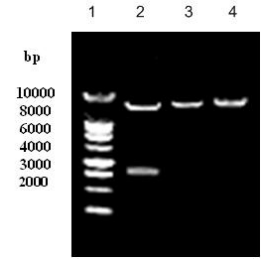
**Restriction mapping of recombinant RevTRE-p53 and RevTet-Off plasmids**. The pRevTRE-p53 plasmid was cleaved with *Hind *III and *Cla *I. The fragments were as follows: Lane 1: 1 Kb marker, Lane 2: the fragments of the pRevTRE vector (6.5 Kb) and the p53 fragment (2.1 Kb). Lane 3: circled pRevTet-Off (7.8 Kb); Lane 4: circled pRevTRE-p53 (8.6 Kb).

The sizes of pRevTet-Off, pRevTRE, the recombinant pRevTRE-p53 plasmid and the cDNA fragment of p53 were 7.8 Kb, 6.5 Kb, 8.6 Kb and 2.1 Kb, respectively. The RevTRE-p53 plasmid was cleaved with *Hind III *and *Cal *I and fragments of 2.1 Kb and 6.5 Kb were generated. Restriction enzyme mapping showed that the full-length p53 cDNA had been successfully cloned into the *Hind *III/*Cla *I sites in the pRevTRE vector, and the sizes of the pRevTet-Off and pRevTRE-p53 fragments were consistent with expectation (Fig. 1).

#### Analysis of recombinant RevTRE-p53 plasmid sequence

To confirm the sequence and orientation of the inserted p53 cDNA in the pRevTRE vector, the purified recombinant pRevTRE-p53 plasmid was verified by sequencing. The p53 insert contained an ATG initiation codon and an entire open reading frame coding for the p53 protein. Sequencing results suggested that the inserted cDNA was identical to the human p53 gene published by GenBank (Accession number: X54156).

#### Selection of high titer packaging cell clones

After transfection with the purified RevTet-Off, RevTRE-p53 and RevTRE-Luc plasmids, PT67 cells were selected with G418 or hygromycin for two weeks. The antibiotic-resistant clones were individually isolated with cloning discs. The viral titers were determined by infecting Hep3B cells with 6 × 10-fold serial diluted virus-producing media from the PT67 clones and showed 5 × 10^4^, 3 × 10^4^, and 6 × 10^4 ^colony forming units for RevTet-Off, RevTRE-p53 and RevTRE-Luc, respectively. The highest titer clones were selected for further experiments.

#### Selection-stable expression of Hep3B/RevTet-Off clones through transient infection by RevTRE-Luc virus

**Figure 2:**
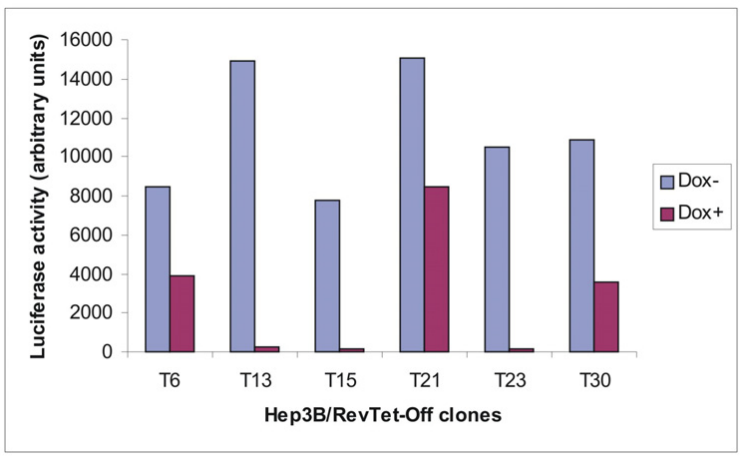
**Doxycycline-regulated luciferase activity in Hep3B/RevTet-Off clones**. Hep3B/RevTet-Off clones were infected transiently using RevTRE-Luc retroviral particles. Cells were cultured in either the presence or absence of 1 μg/ml doxycycline. Clones were assayed by the luciferase assay system.

In an effort to establish a tet-regulated inducible cell line, the RevTet-Off vector was first introduced into Hep3B cells (Hep3B/RevTet-Off) by retroviral infection. The G418-resistant clones were then infected transiently with RevTRE-Luc virus packaged by the PT67 cells. In 32 of the Hep3B/RevTet-Off clones the inductive efficacy of doxycycline-regulated luciferase expression varied from 2 to 70 fold. Six clones showed higher levels of luciferase expression in the absence of doxycycline but different backgrounds in the presence of 1 μg/ml Dox (Fig. 2). The results showed that doxycycline exerted tight control over the expression of this reporter gene in clones T13, T15 and T23. Thus, we selected these clones for further experiments.

#### Identification of doubly-stable expression in Hep3B/RevTet-Off/TRE-p53 clones

**Figure 3:**
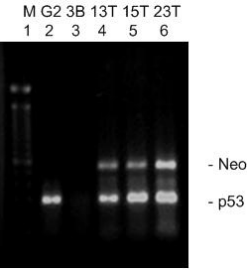
**Establishment of a cell line with doubly-stable expression**. Total RNA was extracted from the selected hygromycin-resistant clones in the absence of doxycycline. Two pairs of primers were used to probe the specific neomycin (Neo^Γ^) and p53 sequences in the tested cells. Lane 1:100 bp marker; Lane 2: HepG2 cells (carrying the wt p53 gene); Lane 3: Hep3B cells (deletion of p53 gene); Lanes 4, 5, and 6: the tested 13T, 15T and 23T clones.

The three selected Hep3B/RevTet-Off clones, T13, T15 and T23, were infected with supernatant from PT67 cells containing the recombinant RevTRE-p53 virus. Hygromycin-resistant Hep3B/RevTet-off/TRE-p53 (Hep3B/tet/p53) clones, 13T, 15T and 23T, were isolated and propagated. We used RT-PCR to establish whether these clones exhibited double-stable expression. Two sets of exploring primers were used to check if these clones are expressing both the neomycin resistance (Neo^r^) gene and p53 gene. The RT-PCR results confirmed expectation (Fig. 3). Therefore, a cell line with doubly-stable gene expression, Hep3B/tet/p53, was established.

#### Selection of a tightly doxycycline-regulated p53-expression clone

**Figure 4:**
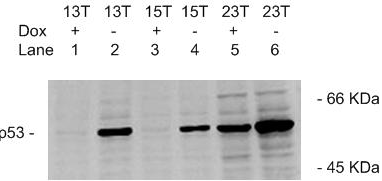
**Selection of the optimal clone under Dox control**. For each of the hygromycin-resistant clones, cells were grown for 24 h in the presence (Dox+) or absence (Dox-) of 1 μg/ml Dox. The expression of p53 protein was analyzed by Western blotting. Lanes 1, 2: 13T (Dox+, Dox-); Lanes 3, 4: 15T (Dox+, Dox-); Lanes 5, 6: 23T (Dox+, Dox-).

**Figure 5:**
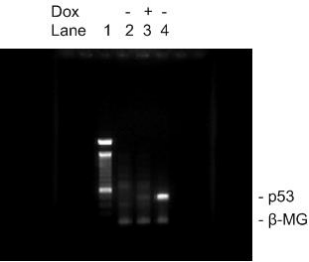
**Semi-quantitative RT-PCR analysis of expression of p53 regulated by Dox**. Hep3B/tet/p53-13 (p53-13) cells were cultured for 24 h in the presence or absence of 1 μg/ml Dox and analyzed by semi-quantitative RT-PCR. Lane 1:100 bp marker; Lane 2: Hep3B cells; Lane 3: p53-13 + 1 μg/ml Dox; lane 4: p53-13 + 0 μg/ml Dox. β-microglobulin (MG) was used as an internal control.

To obtain a high expression, low background clone, 28 different 13T, 15T and 23T clones were analyzed by Western blotting. Some, such as the 23T clone, displayed higher p53 expression in the absence of doxycycline and higher background in its presence, but others, such as the 15T clone, displayed the opposite results. Among the clones tested we found one (13T) that was optimal, exhibiting high p53 expression and low background controlled by doxycycline; this clone was named Hep3B/tet/p53-13 (or p53-13) (Fig. 4). Semi-quantitative RT-PCR demonstrated that the induction of p53 in the Hep3B/tet/p53-13 cells was regulated by doxycycline at the transcriptional level (Fig. 5).

#### Effect of Doxycycline on Hep3B/tet/p53 cell growth

The effect of doxycycline on the growth of Hep3B/tet/p53 cells was examined by trypan blue exclusion. The result showed 1 μg/ml doxycycline had no effect on the growth of Hep3B/tet/p53-13 cells when compared to parental Hep3B cells (result not shown).

#### Regulation of p53 expression by doxycycline

**Figure 6:**
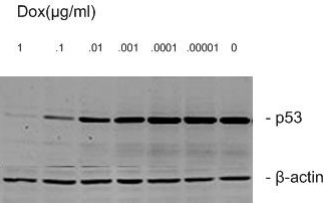
**Expression of p53 protein by Dox controlled quantitatively**. After p53-13 cells were cultured in 0 and 1, 0.1, 0.01, 0.001, 0.0001, 0.00001 μg/ml Dox for 24 h, expression of p53 protein was examined by Western blotting. β-actin was served as an internal control.

**Figure 7:**
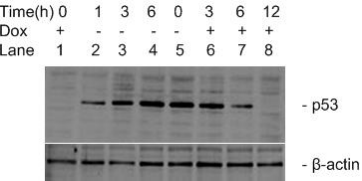
**Time-dependent Dox-regulated expression of p53 protein**. In the removing group, medium was removed 24 h after p53-13 cells were cultured in 1 μg/ml Dox (Dox+), and then the cells were cultured continually in the absence of Dox (Dox-). In the adding group, cells were cultured in the absence of Dox for 24 h, and then the cells were incubated with 1 μg/ml of Dox. The expression of p53 protein was analyzed by Western blotting. Lane1: Dox+; Lanes 2, 3, 4: Dox- 1 h; 3 h; and 6 h; Lane 5: Dox-; Lanes 6, 7, 8: Dox+ 3 h; 6 h; and 12 h. β-actin was served as an internal control.

Hep3B/tet/p53-13 cells were grown in different concentrations of doxycycline and the expression of p53 protein was analyzed by Western blotting. The results showed that 1- 0.00001 μg/ml doxycycline correlated negatively with the level of p53 protein (Fig. 6). Therefore, the expression of p53 protein was regulated by doxycycline in a dose-dependent manner and was almost completely suppressed by a 1 μg/ml concentration. A time-course analysis of this regulation was performed by Western blotting. The results showed that p53 protein expression was gradually enhanced by the removal of doxycycline and reached maximum 6 h after removal of the antibiotic (Fig. 7). In contrast, the protein expression decreased slowly with the addition of doxycycline, becoming almost undetectable 12 h after treatment (Fig. 7). Obviously, doxycycline regulated p53 protein expression in a time-dependent manner.

## Discussion

The recent development of tetracycline (tet)-regulated gene expression has multiplied the tools available for quantitative and temporal control of exogenous genes in different areas of biology and medicine. The first tet-regulated gene expression system (tTA based) [8] consisted of two separate plasmids: the regulatory plasmid for the tet-transactor (tTA) and the response plasmid for the tet-operator minimal promoter driving the gene of interest. In practice, the regulatory plasmid must first be transfected into the target cells. However, conventional transfection methods are not always efficient enough for transfer and high expression of tTA is toxic to cells, probably because of transcriptional "squelching" by the VP16 transactivator domain in tTA [19]. Once cells that stably express tTA have been established, the response plasmid containing the gene of interest under the control of the tet-operator minimal promoter must be introduced. The establishment of a cell line with inducible gene expression can be tedious and time-consuming and modifications have been introduced to circumvent some of the limitations of this system [20-22]. One such modification involves utilizing retrovirus-mediated gene transfer instead of plasmid DNA transfection, thus ensuring more efficient transduction of mammalian cells in vitro. Retroviral vectors for regulated expression are powerful tools for gene transfer since the retroviruses generally integrate as single copies into the target cell genome. Moreover, they are stable, need minimal effort to prepare, and generate populations of cells that can regulate expression of the gene of interest within weeks.

To elucidate the functions of p53 in HCC we chose a retrovirus-mediated tet-regulated gene expression system, RevTet-Off, as a tool. In the present study a full-length cDNA of wt p53 from a human hepatoblastoma cell line, HepG2, which expresses small amounts of wt p53, was cloned into a retroviral response vector, pRevTRE. Subsequently, the target cells, Hep3B, which are derived from a human HCC and have a homozygous deletion of the p53 gene, were first infected by the pRevTet-Off regulatory virus and selected by an antibiotic, G418. Three of 32 tested Hep3B/RevTet-Off clones with the highest maximal luciferase activities were then infected with the recombinant pRevTRE-p53 response virus. After screening with hygromycin, the inducible, doubly-stable p53-expression clones, Hep3B/RevTet-Off/TRE-p53 (Hep3B/tet/p53), were identified by RT-PCR. In 28 other hygromycin-resistant clones we found that the expression of p53 protein was quite different. Clone 23T displayed high levels of p53 protein in the absence of doxycycline, but there was a high basal expression in the presence of 1 μg/ml doxycycline, indicating a high background. In contrast, clone 15T had a low level of p53 protein and low background. In contrast to clones 15T and 23T, clone 13T displayed a higher expression level of p53 and lower background, as demonstrated by Western blot analysis and semi-quantitative RT-PCR.

We speculate that such variations of p53 expression might result from the integration of the retrovirus. In general, the site of integration into the host cell genome is random [23]. When pRevTRE-p53 retroviruses integrate adjacent to an endogenous enhancer element in the Hep3B cell genome, this enhancer could activate p53 transcription even in the presence of 1 μg/ml doxycycline, resulting in a high background. At the same time, the tTA of pRevTet-Off retroviruses might co-operate with this enhancer in the absence of doxycycline to induce quite a high level of p53 expression (as in clone 23T). However, if pRevTRE-p53 retroviruses integrate into a site that is not influenced by an endogenous enhancer, the basal expression level of p53 could be very low as in clone 15T. No methods are currently available for ensuring that a retrovirus integrates at the most useful site. Therefore, it is important to screen the hygromycin-resistant clones for the one that is optimal in terms of high inducible expression and low background.

In this study, an HCC cell line, Hep3B/tet/p53, was successfully established with a retrovirus-mediated tet-regulated gene expression system, RevTet-Off, for inducible, doubly-stable expression of the wt-p53 gene *in vitro*. This cell line is totally different from other lines established through adenovirus-mediated transfer of the p53 gene [24,25]. To the best of our knowledge, such a p53 expression and regulation system has not been reported in a HCC cell line. The system of p53 expression we have established can be controlled by doxycycline not only in turn-on or turn-off manner, but also in a quantitative and temporal manner. The HCC line equipped with pRevTRE-p53 established here can be used not only to study biological effects of the p53 gene *in vitro*, but also in human gene therapy trials in the future.

It is important to investigate the translation product of the gene of interest for its functional role in cell biology or in therapeutic situations when this product is toxic or needs to be maintained at appropriate levels. An inappropriately high level of p53 may become toxic before p53 is delivered to target cells [26]. The pRevTRE-p53 system we have established may overcome this problem since the activation of p53 expression can be controlled quantitatively and temporally by administration of doxycycline.
